# Evaluation of the drug-drug interaction potential of the novel hepatitis B and D virus entry inhibitor bulevirtide at OATP1B in healthy volunteers

**DOI:** 10.3389/fphar.2023.1128547

**Published:** 2023-04-06

**Authors:** Vanessa Zhu, Jürgen Burhenne, Johanna Weiss, Mathias Haag, Ute Hofmann, Matthias Schwab, Stephan Urban, Gerd Mikus, David Czock, Walter E. Haefeli, Antje Blank

**Affiliations:** ^1^ Department of Clinical Pharmacology and Pharmacoepidemiology, Heidelberg University Hospital, Heidelberg, Germany; ^2^ German Center for Infection Research (DZIF) Partner Site Heidelberg, Heidelberg University Hospital, Heidelberg, Germany; ^3^ Dr. Margarete Fischer-Bosch-Institute of Clinical Pharmacology, Stuttgart, Germany; ^4^ University of Tübingen, Tübingen, Germany; ^5^ Departments of Clinical Pharmacology and of Biochemistry and Pharmacy, University of Tübingen, Tübingen, Germany; ^6^ Cluster of Excellence iFIT (EXC2180), Image‐guided and Functionally Instructed Tumor Therapies, University of Tübingen, Tübingen, Germany; ^7^ Department of Infectious Diseases, Molecular Virology, Heidelberg University Hospital, Heidelberg, Germany

**Keywords:** bulevirtide, myrcludex B, hepatitis D virus infection, drug-drug interaction (DDI), SLCO1B, OATP1B, SLC10A1, NTCP

## Abstract

**Introduction:** Bulevirtide is a first-in-class antiviral drug to treat chronic hepatitis B/D. We investigated the drug-drug interaction potential and pharmacokinetics of high-dose subcutaneous bulevirtide (5 mg twice daily) with organic anion transporting polypeptide 1B1 (OATP1B1) and cytochrome P450 (CYP) 3A4.

**Methods:** This was a single-center, open-label, fixed-sequence drug-drug interaction trial in 19 healthy volunteers. Before and at bulevirtide steady state, participants ingested a single 40 mg dose of pravastatin. A midazolam microdose was applied to quantify CYP3A4 activity.

**Results:** At bulevirtide steady state, pravastatin area under the concentration-time curve (AUC_0–∞_) increased 1.32-fold (90% CI 1.08-1.61). The 5 mg bulevirtide twice-daily treatment resulted in a mean AUC_0-12_ of 1210 h*ng/ml (95% CI 1040-1408) and remained essentially unchanged under the influence of pravastatin. CYP3A4 activity did not change to a clinically relevant extent. As expected, total bile acids increased substantially (35-fold) compared to baseline during bulevirtide treatment. All study medication was well tolerated.

**Discussion:** The study demonstrated that high-dose bulevirtide inhibited OATP1B-mediated hepatic uptake of the marker substrate pravastatin but the extent is considered clinically not relevant. Changes in CYP3A4 activity were also not clinically relevant. In conclusion, this study suggests that OATP1B substrate drugs as well as CYP3A4 substrates may safely be used without dose adjustment in patients treated with bulevirtide. However, in patients using high statin doses and where concomitant factors potentially further increase statin exposure, caution may be required when using bulevirtide.

## 1 Introduction

Worldwide, approximately 296 million people suffer from chronic hepatitis B (CHB) infection and 5%–13% of them are coinfected with the hepatitis D virus (HDV) (Chen et al., 2019; Miao et al., 2020), causing accelerated progression of liver disease (World Health Organization, 2021a; World Health Organization, 2021b; Urban et al., 2021). Current treatment options for CHB with HDV coinfection include *off-label* use of interferon alpha, which modulates immune responses against hepatitis B (HBV) and HDV and shows a direct antiviral effect on HDV cell-division mediated spread, and nucleoside/nucleotide analogues such as tenofovir and entecavir to control HBV replication (Abbas and Abbas, 2015; Almeida et al., 2021; Urban et al., 2021; Zhang et al., 2022). However, these drugs rarely result in cure and permanent seroconversion (Tang et al., 2014; World Health Organization, 2015; Farci and Anna Niro, 2018; World Health Organization, 2021b; Urban et al., 2021). Until 2020, there was no approved treatment for chronic hepatitis D (CHD). Thus, the approval of the new HBV and HDV entry inhibitor bulevirtide in July 2020 for the treatment of chronic HDV infection was a milestone providing the first treatment option for CHD patients, which may - in optimal time and dosing - even be curative, which has however not yet been shown (European Medicines Agency, 2020; Almeida et al., 2021; Urban et al., 2021). The approval of the FDA is still awaited.

Bulevirtide consists of a myristoylated peptide containing 47 amino-acids derived from the preS1-domain of the hepatitis B virus (HBV) L-surface protein. Bulevirtide acts as a virus entry inhibitor by binding to and inactivating the hepatic sodium taurocholate co-transporting polypeptide (NTCP, SLC10A1), which is the natural bile acid transporter and also promotes entry of HBV and HDV into hepatocytes (Schulze et al., 2010; Yan et al., 2012; Ni et al., 2014). Bulevirtide blocks the physiological function of NTCP with an IC_50_ at 50 nM (Nkongolo et al., 2014), total bile acids in plasma consecutively increase by more than 19-fold, specific conjugated bile acids, such as taurocholic acid, increase by over 100-fold. (Blank et al., 2018). Bulevirtide as a peptide is most likely degraded by proteases into amino acids, there is no indication that cytochrome enzymes are involved in the metabolism of bulevirtide. The IC_50_ to inhibit the HBV/HDV entry is in the sub-nanomolar range (Schulze et al., 2010).

Several clinical trials with bulevirtide showed an excellent safety profile and a significant reduction of biomarkers such as HDV RNA in serum and liver and normalization of alanine aminotransferase (ALT) in patients with CHD (Blank et al., 2016; Bogomolov et al., 2016; Blank et al., 2018; Wedemeyer et al., 2019; European Medicines Agency, 2020; Wedemeyer et al., 2022a; Wedemeyer et al., 2022b). In addition, elevated bile acids did not cause any adverse clincal symptoms and showed no long-term effects on relevant cardiovascular biomarkes (Blank et al., 2016; Bogomolov et al., 2016; Blank et al., 2018; Wedemeyer et al., 2019; Wedemeyer et al., 2022b; Stoll et al., 2022).

The clinical effect of inhibiting HDV entry is clearer than the inhibition of the HBV entry, where trials failed to show effects on markers of HBV infection. (Bogomolov et al., 2016; Wedemeyer et al., 2022b).

*In vitro*, bulevirtide not only inhibits NTCP but also the organic anion transporting polypeptide 1B1 (OATP1B1) at nanomolar concentrations (IC_50_ = 530 (±90) nM) and to a lesser extent OATP1B3 (IC_50_ = 8650 (±3740) nM) (Blank et al., 2017). Treatment with 10 mg daily s. c. bulevirtide results in peak plasma concentrations at steady state of approximately 80 nM, a single infusion of 20 mg bulevirtide resulted in a 520 nM peak concentration (Blank et al., 2016; Blank et al., 2018). Although the currently conditionally licensed dose in Europe for patients is 2 mg s. c. per day, the optimal therapeutic dose is still under evaluation. Therefore, potential OATP inhibition needs further evaluation to clarify the drug interaction potential with higher doses (Lampertico et al., 2022).

As a liver-specific uptake transporter, OATP1B contributes to the targeting of many substrate drugs from different classes and most importantly lipid-lowering statins, whose adverse events are closely linked to drug-drug interactions (Niemi et al., 2011). We assessed bulevirtide’s interaction potential with the OATP1B marker substrate pravastatin (Food and Drug Administration, 2020). Pravastatin is proposed by the FDA as a marker substrate for drug interaction trials to evaluate OATP1B1 and 1B3, it does not undergo relevant cytochrome (CYP) P450 metabolism (Neuvonen et al., 1998; Jacobsen et al., 1999; Jacobson, 2004) and is mainly excreted unchanged (Williams and Feely, 2002; Neuvonen et al., 2008). The agency recommends pravastatin as OATP1B marker substrate although their table mentions it to also being substrate to other transporters especially NTCP (Food and Drug Administration, 2020). We cannot rule out that effects of the NTCP blockade through bulevirtide may also have an impact on its role as marker for OATP, however preclinical data remain controversial and clinical data do not confirm the involvement of NTCP for pravastatin transport (Kivisto and Niemi, 2007; Choi et al., 2011; Lu et al., 2016; Wang et al., 2020).

We aimed to also enroll participants with genetically impaired OATP1B transporter function, such as carriers of the SLCO1B1*5 and SLCO1B1*15 haplotype (Kalliokoski and Niemi, 2009; Niemi et al., 2011; Nakanishi and Tamai, 2012), which might affect the individual extent of a drug-drug interaction. In addition, we evaluated the pharmacokinetic and pharmacodynamic effects of twice-daily dosing with 5 mg s. c. bulevirtide.

## 2 Participants and methods

### 2.1 Ethical and legal aspects

This trial was carried out in accordance with the principles of the Declaration of Helsinki, the standards of Good Clinical Practice as outlined by the International Council for Harmonization of Technical Requirements for Pharmaceuticals for Human Use, and all legal requirements for clinical trials in Germany. It was approved by the responsible Ethics Committee of the Medical Faculty of Heidelberg University (AFmo-273/2018; June 20, 2018) and the competent national authority (BfArM, Bonn, Germany, EudraCT: 2018-000012-21; July 4, 2018). All participants provided written informed consent prior to any trial-related procedure.

### 2.2 Trial design

The trial was carried out between 19/07/2018 and 23/11/2018 at the Clinical Research Unit of the Department of Clinical Pharmacology and Pharmacoepidemiology (Heidelberg University Hospital, Germany, certified according to DIN EN ISO 9001:2015). We conducted a single-centre, open-label, fixed-sequence drug-drug interaction trial in healthy volunteers. The primary objective was to evaluate the effect of a 5 mg dose of bulevirtide, given twice a day (bid) and dosed to steady state on the pharmacokinetics of a single dose of 40 mg pravastatin (sampling over 24 h). The dose of bulevirtide was 5 times higher than the currently conditionally approved daily dose of 2 mg, however it was chosen to serve as a worst-case scenario in a situation where the optimal therapeutic dose remains unclear. Secondary objectives were the evaluation of steady-state pharmacokinetics of bulevirtide with a 5 mg bid regimen (evaluation over the dosing interval 0–12 h), the effect of pravastatin on bulevirtide pharmacokinetics (evaluation over the dosing interval 0–12 h), the pharmacodynamic effects on plasma bile acid concentrations at bulevirtide steady state (evaluation over 0–24 h at baseline, evaluation over the bulevirtide dosing interval 0–12 h at bulevirtide steady-state, and after end of treatment 0–72 h), and the effect of both drugs on CYP3A4 activity as assessed by administration of an oral 30 µg midazolam microdose suing a limited sampling strategy (evaluation over the dosing interval 2–4 h) (Katzenmaier et al., 2010; Katzenmaier et al., 2011). Additionally, the pharmacokinetics in volunteers with genetic polymorphisms of SLCO1B was investigated.

**FIGURE 1:**
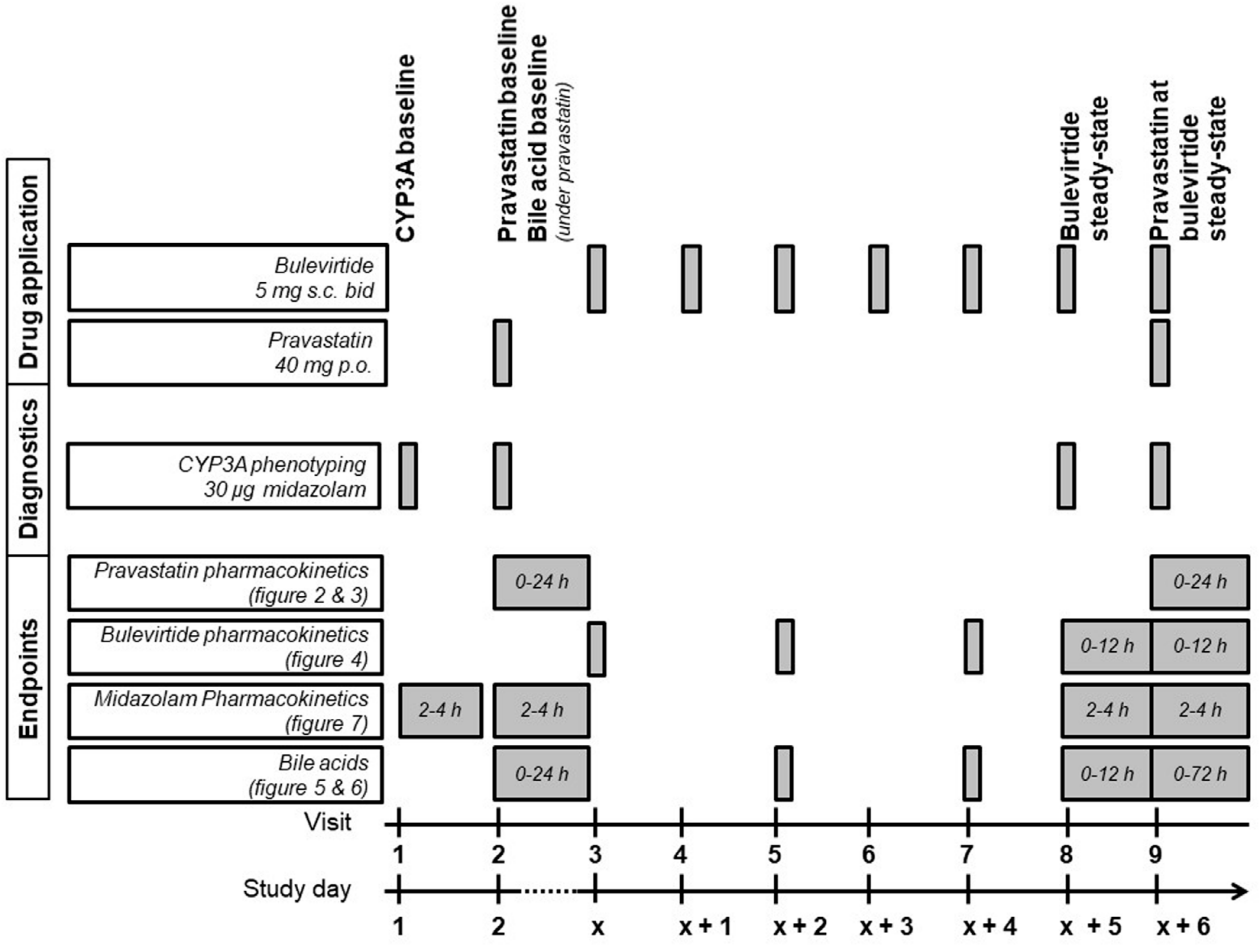
Trial flowchart of the treatment period (bid, twice a day; CYP3A, cytochrome P450 3A; p. o., per os; s. c., subcutaneously).

The detailed trial flowchart is presented in Figure 1. Participants were dosed at the site with 5 mg subcutaneous (sc) bulevirtide (Baccinex SA, Courroux, Switzerland) bid for 7 d. Based on the previously reported pharmacokinetics a bulevirtide steady state was assumed on day 6 of treatment (Blank et al., 2018). The pharmacokinetics of a single 40 mg dose of pravastatin (Pravastatin HEXAL^®^, Hexal, Holzkirchen, Germany) was assessed at baseline prior to starting bulevirtide and at bulevirtide steady state.

CYP3A4 activity was determined at baseline, during a single dose of 40 mg pravastatin, and at bulevirtide steady state before and during a single dose of 40 mg pravastatin. The CYP3A4 activity was assessed by administering an oral 30 µg midazolam microdose (Dormicum^®^ V 5 mg/5 ml solution for injection, Cheplapharm Arzneimittel GmbH, Greifswald, Germany) diluted in 100 ml water, following an established procedure (Katzenmaier et al., 2010; Katzenmaier et al., 2011). Midazolam was administered 30 min after the bulevirtide injection. Bulevirtide pharmacokinetics were evaluated at steady state without and with concurrent pravastatin dosing (30 min after bulevirtide injection). Bile acid profiles were evaluated at baseline and at bulevirtide steady state with and without pravastatin. Because bile acids are influenced by food intake, all participants started fasting and received comparable meals at predetermined times after drug administration on the days of the pharmacokinetic assessments.

A group of 20 healthy volunteers between 18 and 50 y was enrolled of whom at least twelve had to be homozygous or heterozygous carriers of the wild-type allele SLCO1B1*1A (Nakanishi and Tamai, 2012). Participants were healthy as assessed by physical and mental health examination (including medical history, physical assessments, vital signs, and electrocardiogram) and laboratory evaluation. Females of childbearing potential were required to have negative serum pregnancy tests at screening and all participants had to be willing to use a highly effective method of contraception during treatment and for 3 months after the last administration of the investigational drugs. The main exclusion criteria contained any acute or chronic illness, use of an investigational drug within 30 d prior to trial start, haemoglobin <12 g/dL (male) or <11 g/dL (female), history of cholecystectomy, history of severe allergic reactions, history of any form of hepatitis (except hepatitis A), a positive human immunodeficiency virus antibody screen, a positive drug screen, and regular intake of alcohol. Participants were not allowed to take any medication (except hormonal contraception) or to consume citrus fruits and methylxanthine-containing foods and beverages within 7 d prior to trial start and throughout the trial. Alcohol was not allowed within 24 h prior to trial start and throughout the trial. A full list of the applied inclusion and exclusion criteria is provided in the Supplementary Material.

### 2.3 Genotyping

Genotyping of SLCO1B1 polymorphisms was carried out by a LightCycler 480-based method with hybridization probes as previously described determining the two single nucleotide polymorphisms c.521T>C (V174A, rs4149056) and c.388A>G (N130D, rs2306283) (Aklillu et al., 2011). The combination results defined the genotypes SLCO1B1*1A (c.521T, c.388A), *1B (c.521T, c.388G), *5 (c.521C, c.388A), and *15 (c.521C, c.388G). Homozygous carriers of SLCO1B1*1A represent the wildtype genotype and were used for comparisons with genotypes expected to have a reduced transport function. Heterozygous SLCO1B1*1A as well as homozygous or heterozygous *1B (rs2306283) carriers are not expect to have an altered transport function. Genetically reduced transporter function was expected for carriers of the SLCO1B1*5 and SLCO1B1*15 haplotype (Kalliokoski and Niemi, 2009; Niemi et al., 2011).

### 2.4 Bioanalytical procedure

Blood samples for the quantification of bulevirtide, pravastatin, midazolam, and bile acid profiles were taken before and at multiple timepoints up to 48 h after drug intake. Samples were centrifuged at 3600 g and 4°C for 10 min, and plasma was separated and stored at ≤ −20°C until analysis. Backup samples were stored at −80°C. Bulevirtide plasma concentrations were determined by ultra-performance liquid chromatography coupled to tandem mass spectrometry (UPLC-MS/MS) as described previously (Sauter et al., 2021). Pravastatin plasma concentrations were quantified by liquid chromatography-tandem mass spectrometry (LC-MS/MS) similar to a method described elsewhere (Thiel et al., 2015). Briefly, plasma was spiked with internal standard [2H3] pravastatin, extracted with diethyl ether as described in (Igel et al., 2006) and subsequently analyzed with LC-MS/MS (Thiel et al., 2015). The quantification of midazolam plasma concentration was based on an previously reported UPLC-MS/MS assay (Burhenne et al., 2012). Plasma concentrations of bile acids were analyzed to confirm the expected pharmacodynamic effect of bulevirtide, the bile acid elevation. Bile acids were therefore only measured in a randomly selected sample-set of ten participants. Bile acids were analysed by liquid chromatography quadrupole time-of-flight mass spectrometry as described before (Haag et al., 2015). Total bile acids were determined as the sum of unconjugated (cholic acid, ursodeoxycholic acid, chenodeoxycholic acid, deoxycholic acid, and lithocholic acid), glycine-conjugated (glycoursodeoxycholic acid, glycocholic acid, glycochenodeoxycholic acid, glycodeoxycholic acid, and glycolithocholic acid), and taurine-conjugated (tauroursodeoxycholic acid, taurocholic acid, taurochenodeoxycholic acid, taurodeoxycholic acid, and taurolithocholic acid) bile acids. Method validations were performed according to the Guideline on Bioanalytical Method Validation published by the European Medicines Agency and/or the US Food and Drug Administration (European Medicines Agency, 2011; US Department of Health and Human Services, 2018).

### 2.5 Pharmacokinetic and statistical analyses

For pravastatin and bulevirtide pharmacokinetic analyses standard noncompartmental pharmacokinetic parameters were determined using Phoenix WinNonlin version 8.2 (Certara, St. Louis, MO, United States). Maximum plasma concentration (C_max_) and time to reach C_max_ (T_max_) were directly obtained from pharmacokinetic data. Area under the concentration time curve from 0 to 12 h (AUC_0–12_; bulevirtide, bile acids) was determined using the predose time point until the last observed timepoint at 12 h. For AUC_0–∞_ (pravastatin), AUC was determined using the log-linear trapezoidal rule and by adding the extrapolated part until infinity. Half-life (t_1/2_) was calculated as $\frac{\ln\left(2\right)}{\lambda z}$ , whereas the elimination rate constant λz was estimated using log-linear regression of the elimination phase. Apparent clearance (Cl/F) was calculated as $\frac{dose}{AUC0-12}$ (bulevirtide) and $\frac{dose}{AUC0-\infty }$ (pravastatin). CYP3A4 activity was determined using a limited sampling strategy and determining the estimated partial metabolic clearance (eCl_met_) (derived from midazolam AUC_2-4_) as described earlier (Katzenmaier et al., 2010). Pharmacokinetic parameters are described as geometric means with 95% confidence interval.

For bile acids, C_max_ and T_max_ were directly obtained from concentration data and AUC_0-12_ was determined using the concentrations at predose time point until the last observed timepoint at 12 h.

Based on the guideline for bioequivalence trials (European Medicines Agency, 2010) changes in exposure were evaluated as follows: A paired t-test on log-transformed variables was applied to evaluate pravastatin pharmacokinetic parameter C_max_ and AUC_0-∞_ under the influence of bulevirtide, and bulevirtide pharmacokinetic parameter C_max_ and AUC_0-12_ at steady state and at steady state under the influence of pravastatin. Geometric mean ratios for AUC and C_max_ were described by using the 90% confidence interval and the significance level of ≤0.1. To evaluate difference for T_max_, Cl/F and t_1/2_ pharmacokinetic log-transformed (except for T_max_) variables were evaluated by t-test using the 95% confidence interval and the significance level of ≤0.5. Midazolam AUC_2-4_, eCl_met_, and AUC_0-12_ of bile acids on four study days were evaluated by analysis of variance with adjustments for multiple testing, using a significance level of ≤0.05.

Statistical analyses and graphs were generated using GraphPad Prism version 9.2 (GraphPad Software Inc., La Jolla, CA, United States, (RRID:SCR_002798)). The extent of the drug interaction in volunteers with SLCO1B1 polymorphisms was evaluated descriptively. In a *post hoc* analysis bulevirtide exposure (AUC_0-24_) with 5 mg sc bid dosing in this trial was compared to exposure with 10 mg sc bulevirtide once daily as observed in a previous trial (Blank et al., 2018), by predicting the AUC_0-24_ by doubling AUC_0-12_, assuming the absence of circadian variations in bulevirtide pharmacokinetics.

## 3 Results

### 3.1 Study population

Twenty healthy volunteers (12 females, 8 males; 18 Caucasians, 1 Hispanic, 1 Arabic) were enrolled in this trial. One volunteer terminated the trial prematurely due to an unrelated urinary tract infection after having received the first microdose of midazolam; The remaining 19 volunteers completed the trial as planned. Their median age was 23 y (range 18–45), their body weight [mean ± standard deviation (SD)] was 69.6 kg (±15.8), body mass index was 23.4 kg/m2 (±3.4), and their estimated creatinine clearance (Cockcroft-Gault formula) was 133 ml/min (±30.2). Participants had the following SLCO1B1 genotypes: *1A/*1A (n = 9, homozygous wild-type group; 1 drop out = 8 evaluable volunteers), *1A/*1B (n = 5, heterozygous wild-type group), *1B/*1B (n = 2), *5/*15 (n = 1), *1B/*15 (n = 1), *1A/*15 or *1B/*5 (n = 1), and *15/*15 (n = 1).

### 3.2 Pharmacokinetic evaluation of the OATP1B marker substrate pravastatin

**FIGURE 2:**
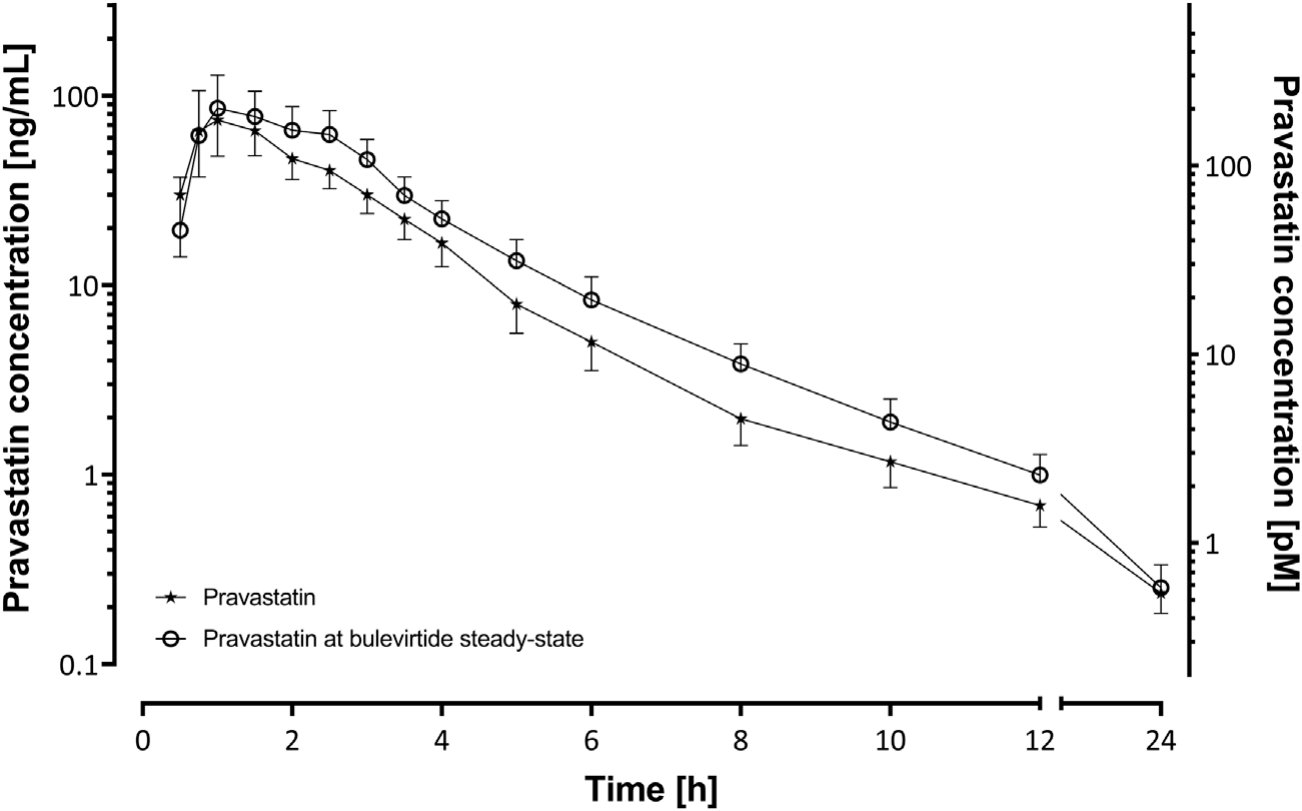
Plasma concentration-time profiles of a 40 mg single oral dose of pravastatin (N = 19) before and at bulevirtide steady state (5 mg, twice daily, subcutaneously; geometric means ±95% confidence interval).

**FIGURE 3:**
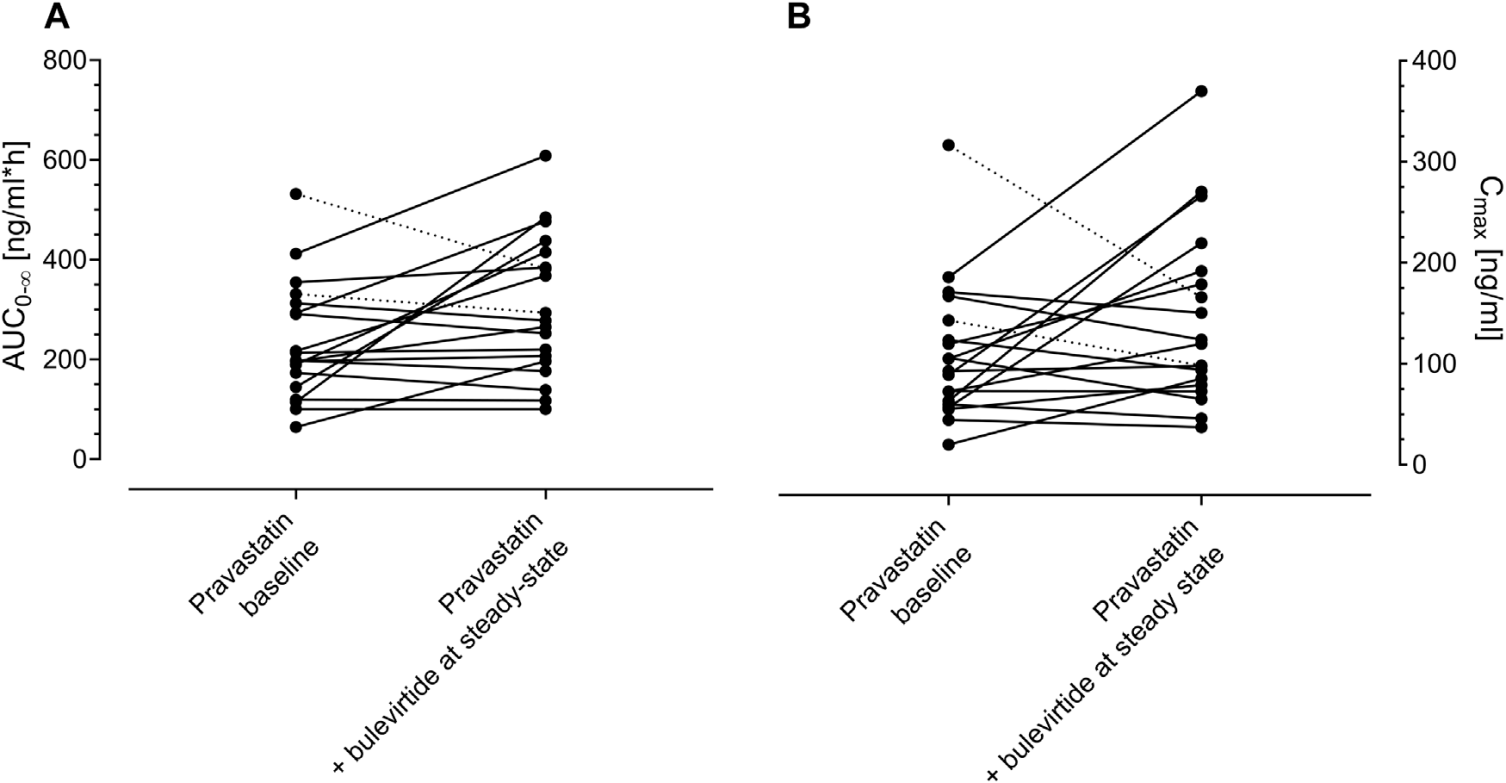
Individual values of pravastatin AUC_0–∞_ **(A)** and C_max_ **(B)** after a 40-mg single oral dose of pravastatin at baseline and at bulevirtide steady state (5 mg, twice daily, subcutaneously, N = 19). Dotted lines indicate the two carriers of genotype *5/*15 and *15/*15. (See also Table 1).

Bulevirtide increased pravastatin geometric mean ratio (GMR) for AUC_0-∞_ to 1.32-fold (90% CI 1.08-1.62, *p* = 0.03) and GMR for C_max_ to 1.32 (1.01–1.73, *p* = 0.09) in the overall trial population (N = 19, Figure 2, Table 1) whereas T_max_ and t_1/2_ did not change (Table 1). Individual changes of the pravastatin AUC_0–∞_ and C_max_ are shown in Figure 3.

**TABLE 1:** Pharmacokinetics of a 40-mg single oral dose of pravastatin alone and at bulevirtide steady state (see also Figure 3).

Pharmacokinetic parameter^a^	Pravastatin		*p*-value
	baseline	at bulevirtide steady state	
AUC_0–∞_ (h*ng/ml)	207 (161; 267)	273 (214; 348)	
*Geometric mean ratio*		*1.32 (1.08–1.62)*	*0.03*
C_max_ (ng/ml)	91.4 (67.8; 123)	121 (89.8; 163)	
*Geometric mean ratio*		*1.32 (1.01–1.73)*	0.02
T_max_ (h)	0.97 (0.80; 1.18)	1.22 (0.97; 1.52)	ns
Cl_/F_ (L/h)	193 (150; 249)	147 (115; 187)	0.03
t_1/2_ (h]	1.98 (1.69; 2.31)	1.84 (1.60; 2.11)	ns

AUC_0–∞_, area under the concentration-time curve extrapolated from time 0 to infinity; C_max_, maximum concentration; Cl_/F_, apparent clearance; ns, statistically not significant; t_1/2_, terminal half-life; T_max_, time to reach C_max_.

^a^ Pharmacokinetic parameter are shown as geometric means with 95% confidence interval (*p* < 0.05 is considered to be statistically significant). Geometric mean ratios for AUC and C_max_ are shown with 90% confidence interval (a *p*-value of 0.1 is considered statistically significant). Geometric mean ratios for for T_max_, CL_/F_, and t_1/2_ are shown with 95% confidence interval (a *p*-value of 0.05 is considered statistically significant). (European Medicines Agency, 2010).

The trial population excluding the two carriers of genotypes associated with reduced transport capacity (*5/*15, (*15/*15) showed a GMR for AUC_0–∞_ of 1.4 (90% CI 1.13–1.73, *p* = 0.02) and a GMR for C_max_ of 1.47 (90% CI 1.03-2.11, *p* = 0.07). Homozygous carrier of SLCO1B1*1A/*1A (N = 8) had comparable AUC values to the overall population at baseline and showed an increase in GMR for AUC_0–∞_ of 1.76 (90% CI 1.17-2.64) and a GMR for C_max_ of 1.88 (90% CI 1.11-3.2). The SLCO1B1*5/*15 and SLCO1B1*15/*15 carriers, i.e., with an assumed decreased transport function, had a relatively high pravastatin exposure at baseline (without bulevirtide) with an AUC_0–∞_ of 331/532 h*ng/ml respectively, and a C_max_ 143/316 ng/ml, respectively. The AUC ratio for these two individuals decreased to 0.89/0.72 respectively (SLCO1B1*5/*15/SLCO1B1*15/*15; See dotted lines Figure 3). Individuals without a wildtype SLCO1B1*1A gene did not show remarkable differences as compared to the overall population, details can be found in the Supplementary Table S1.

### 3.3 Bulevirtide pharmacokinetic evaluation

**FIGURE 4:**
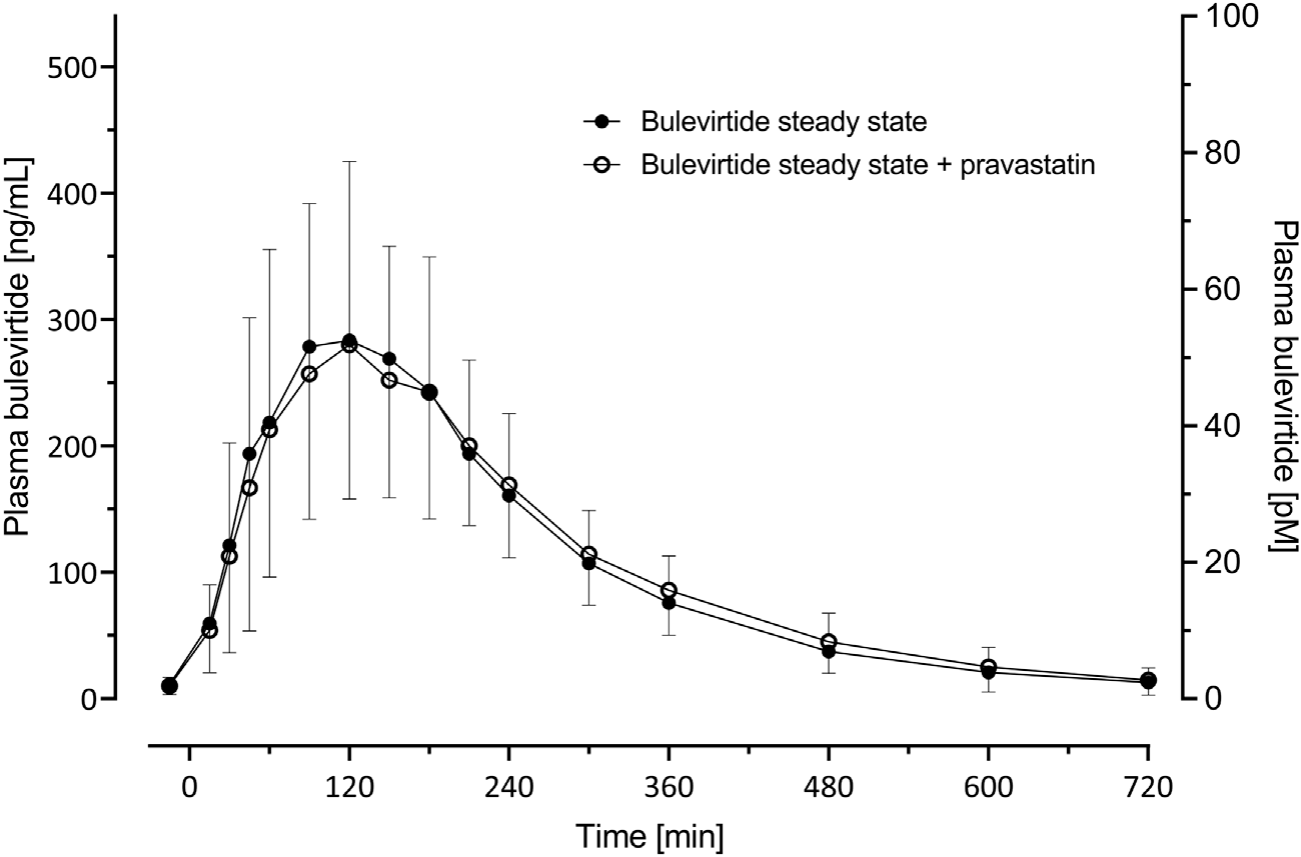
Plasma concentration-time profiles (geometric mean; 95% confidence interval) of bulevirtide at steady-state (5 mg, twice daily, subcutaneously, given 30 min prior to pravastatin) without and with a 40 mg single oral dose pravastatin; N = 19.

Bulevirtide pharmacokinetics were not influenced by co-administration of pravastatin (Table 2; Figure 4); The GMR without and with pravastatin of bulevirtide AUC_0–12_ was 1.01 (90% CI: 0.94-1.09) and of C_max_ was 0.93 (0.84-1.02). In a *post hoc* analysis, the 24-h exposure (AUC_0-24_) in the present trial with 5 mg bulevirtide bid was 29% larger than after the once-daily 10 mg s.c. administration in a previous trial in healthy volunteers (2420 ng/ml*h compared to 1840 ng/ml*h, *p* = 0.01) (Blank et al., 2018).

**TABLE 2:** Bulevirtide pharmacokinetics after subcutaneous administration of 5 mg, twice daily, with and without a single 40 mg dose of pravastatin (see also Figure 4).

Pharmacokinetic parameter^a^	Bulevirtide steady state		*p*-value
	Without pravastatin	With pravastatin	
AUC_0–12_ (h*ng/ml)	1210 (1039; 1408)	1225 (1047; 1433)	
*Geometric mean ratio*		*1.01 (0.94; 1.09)*	ns
C_max_ (ng/ml)	285 (229; 355)	264 (214; 326)	
*Geometric mean ratio*		*0.93 (0.84; 1.02)*	ns
T_max_ (h]	1.77 (1.47; 2.23)	2.12 (1.82; 2.54)	ns
Cl_/F_ (ml/min)	68.9 (59.2; 80.2)	68.0 (58.1; 79.6)	ns

AUC_0–∞_, area under the concentration-time curve extrapolated from time 0 to infinity; C_max_, maximum concentration; ns, statistically not significant; T_max_, time to reach C_max_.

^a^ Pharmacokinetic parameter are shown as geometric means with 95% confidence interval. Geometric mean ratios for AUC and C_max_ are shown with 90% confidence interval (a *p*-value of 0.1 is considered statistically significant). Geometric mean ratios for T_max_ and CL_/F_ are shown with 95% confidence interval (a *p*-value of 0.05 is considered statistically significant). (European Medicines Agency, 2010).

### 3.4 Pharmacodynamic evaluation: bile acids

**FIGURE 5:**
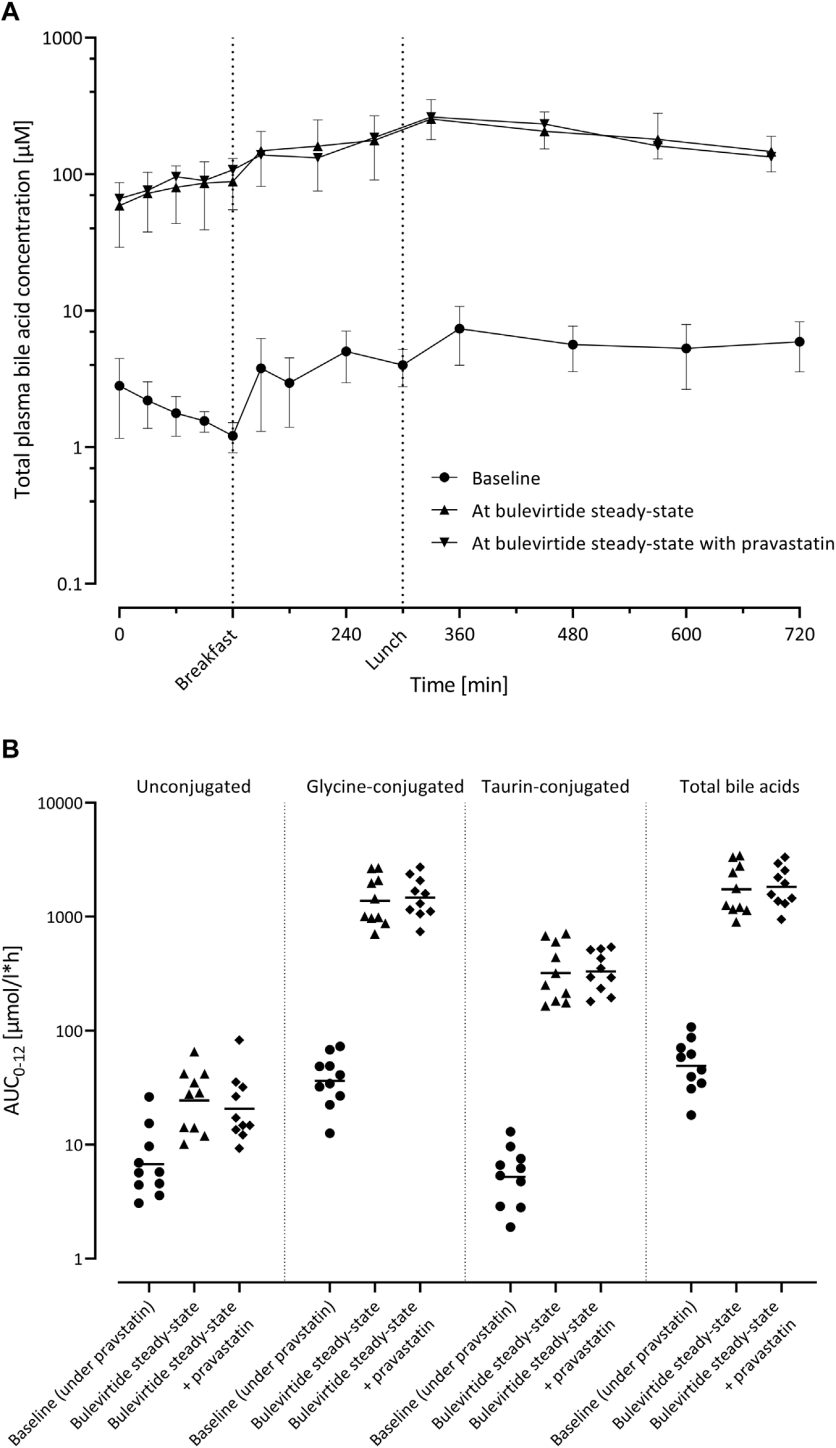
**(A)** Plasma concentration-time profiles of total bile acids (n = 10; geometric mean ±95% confidence interval) before bulevirtide and at bulevirtide steady state (5 mg, twice daily, subcutaneously) with and without a single dose of pravastatin. **(B)** AUC_0–12_ of unconjugated, glycine-conjugated, taurine-conjugated, and total bile acids in ten participants before bulevirtide and at bulevirtide steady state with and without pravastatin. Horizontal lines: geometric means.

Bulevirtide at steady state increased the geometric mean AUC_0–12_ of total bile acids 35.2-fold from 49.8 µmol/l*h (95% CI 34.1; 72.7) at baseline to 1752 µmol/l*h (95% CI 1225; 2506; *p* < 0.0001) unconjugated bile acids 3.62-fold (*p* = 0.001), taurine-conjugated bile acids 61.5-fold, and glycine-conjugated bile acids 38.0-fold (both *p* < 0.0001; Figure 5). After co-administration with pravastatin, comparable values of bile acid AUC_0–12_ were observed (Supplementary Table S1).

### 3.5 CYP3A4 activity

**FIGURE 6:**
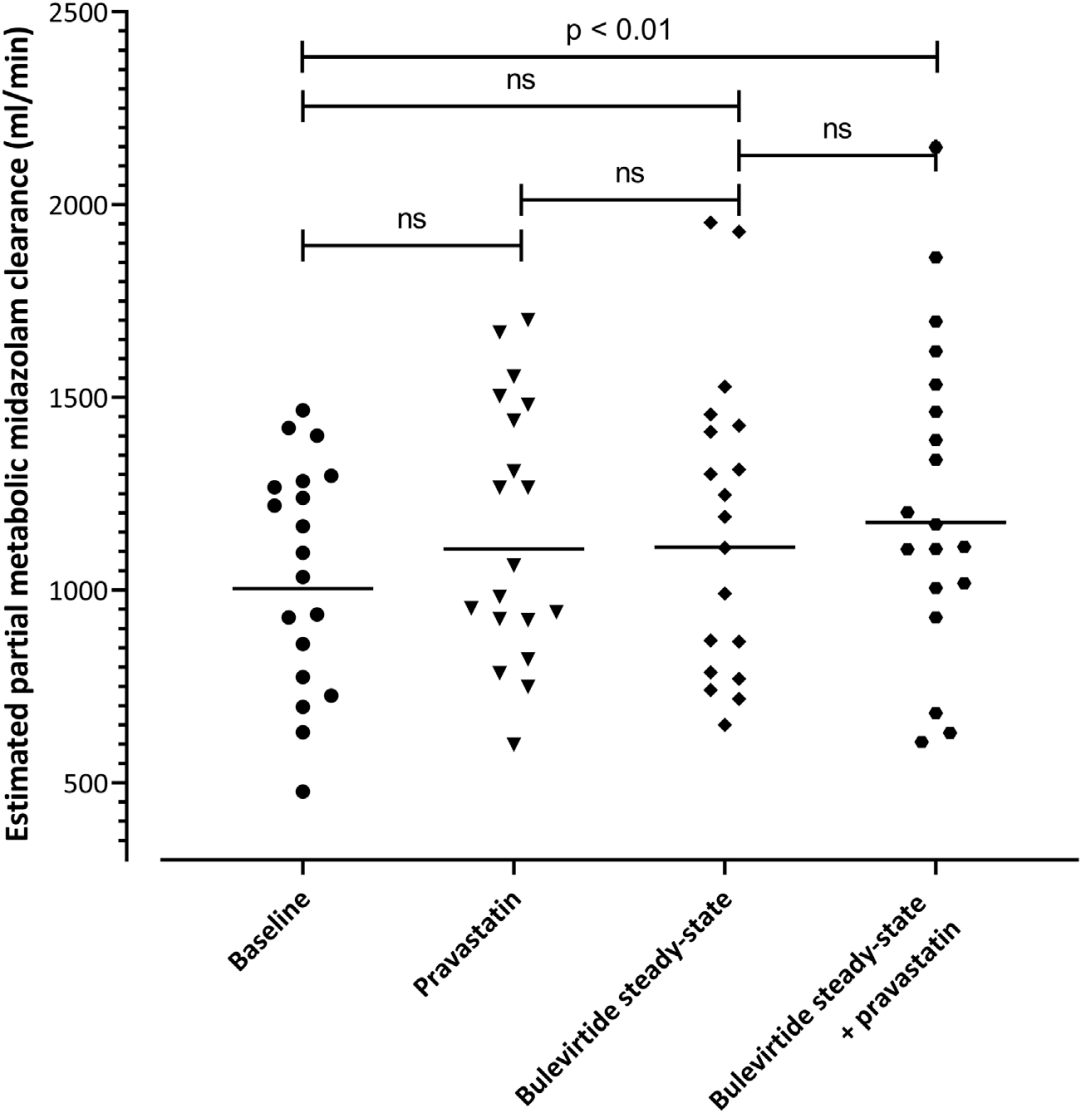
Estimated partial metabolic midazolam clearance at baseline, with a 40-mg single dose of pravastatin, at bulevirtide steady state, and during co-administration of bulevirtide and pravastatin. Horizontal lines: geometric means; ns, not significant.

eCl_met_ of midazolam as marker for CYP3A4 activity was evaluated at 4 time points in the trial. The geometric mean clearance values were 1004 mL/min (95% CI: 863-1169) at baseline, 1107 ml/min (957-1280) with single-dose pravastatin, 1111 ml/min (946-1306) at bulevirtide steady state, and 1176 ml/min (993-1392) at bulevirtide steady state with pravastatin. The corresponding GMR compared to baseline was 1.10 (single dose pravastatin, 95% CI 1.04-1.17), 1.11 (at bulevirtide steady state, 95% CI 1.02-1.20), and 1.17 (at bulevirtide steady state with a single dose of pravastatin, 95% 1.09-1.26). Explorative evaluation considering multiple testing revealed a small, but statistically significant difference (*p* = 0.009) for the GMR at bulevirtide steady state with a single dose of pravastatin (Figure 6).

### 3.6 Safety

All trial drugs were well tolerated; in total, 60 adverse events occurred in 19 of 20 volunteers and all resolved without sequelae. None of them was serious, 58 events were mild and two were of moderate nature according to CTCAE grading. One adverse event led to withdrawal from the trial after trial day 1 due to a urinary tract infection, that was judged as being unrelated to the applied study drug, which was a midazolam microdose. Forty-four adverse events were considered at least possibly related to the trial medication, with injection site reactions (15 events) and headache (8 events) being the most common (Supplementary Table S2).

## 4 Discussion

The mechanism of action of the novel HBV/HDV entry inhibitor bulevirtide is the inhibition of the NTCP receptor function for HBV/HDV uptake into hepatocytes. It has been shown that the uptake of HBV and HDV is inhibited at sub-nanomolar ranges (Schulze et al., 2010), whereas the bile acid transport is in comparable conditions only inhibited at 50 nM (Nkongolo et al., 2014). Currently used therapeutically doses for bulevirtide blocking the physiological bile acid transport increase circulating bile acids by more than an order of magnitude (Blank et al., 2018). In addition, bulevirtide is also an inhibitor of OATP1B1 *in vitro* (Blank et al., 2017). Both transporters play a critical role in the hepatic uptake of bile acids (Slijepcevic et al., 2017; Suga et al., 2017; Toth et al., 2018) and of a variety of drugs. Some xenobiotics are substrates of both transporters (e.g., rosuvastatin (Ho et al., 2006; Lou et al., 2014)), suggesting that there is a functional overlap of the transporters with respect to endogenous and exogenous substrates. Inhibition of these transporters can therefore impair hepatic uptake (and clearance) and thus increase systemic exposure of their substrates, potentially leading to major adverse consequences (Koenen et al., 2011). Finally, it is also conceivable that the considerable accumulation of endogenous substrates (e.g., bile acids) under bulevirtide treatment could competitively inhibit the transport of other substrates.

In the present trial, a new high-dose regimen of 5 mg bulevirtide bid was used. Compared to the steady-state exposure of a once-daily administration of 10 mg, the bulevirtide steady-state exposure with 5 mg bid (total daily dose 10 mg) was 29% larger (Blank et al., 2018). This is not unexpected for a drug which follows a target-mediated drug disposition, where the 10 mg dose was in a modelling approach not expected to provide a target coverage over 24 h (Blank et al., 2016). Concurrently, bile acids increased considerably and the fold-changes of total bile acids after 5 mg bid dosing were also higher (35-fold increase) than after once daily administration of 10 mg bulevirtide (19-fold) (Blank et al., 2018). This seems at a first glance contradictory as the dose should coincide with the level of NTCP-inhibition, however, the longer duration of higher concentrations, sufficient to inhibit NTCP-mediated bile acid uptake over 24 h, may explain this difference.

In this study, the average exposure of the sensitive OATP1B marker substrate pravastatin increased by only 32% with bulevirtide at steady state. Pravastatin clearance is mainly dependent on OATP1B uptake. Although the FDA indicates that pravastatin may also be a substrate of NTCP on their list of marker substrates, to our best knowledge there is no data convincingly showing a major role of NTCP in the elimination of pravastatin. (Kivisto and Niemi, 2007; Choi et al., 2011; Lu et al., 2016; Wang et al., 2020). In this trial we therefore conclude, that the described effect is due to a drug interaction with OATP. However, we cannot completely rule out any role of NTCP on pravastatin disposition. A potential drug interaction by NTCP blockade by bulevirtide might potentially be a minor part of the overall effect. Several clinical interaction trials with potent OATP1B1 inhibitors have already demonstrated the essential role of OATP1B in the disposition of pravastatin. For instance, co-administration of OATP1B inhibitors such as ciclosporin increases pravastatin exposure 5-10-fold, single-dose rifampicin 2.27-fold, gemfibrozil 2-fold (Olbricht et al., 1997; Kyrklund et al., 2003; Hedman et al., 2004; Deng et al., 2009), and boceprevir 1.63-fold (Hulskotte et al., 2013).

*In vitro*, bulevirtide inhibited OATP1B1 and to a lesser extent OATP1B3 (Blank et al., 2017) suggesting that the AUC_0–∞_ increase of 32% for pravastatin during bulevirtide treatment in our trial could indicate a mild, but probably clinically unrelevant *in vivo* inhibition of OATP1B-mediated pravastatin uptake into the liver. On the other hand, especially conjugated bile acids are endogenous substrates of OATP1B (Trauner and Boyer, 2003), their increase under the bulevirtide treatment with NTCP inhibition may also have resulted in a competitive inhibition of pravastatin uptake. Lastly, bulevirtide has a very high degree of plasma protein binding (>99.9%) and thus free concentrations may not be large enough even with a 5 mg BID dose to achieve significant OATP1B1/3 inhibition of pravastatin uptake.

However, our findings show a pravastatin AUC_0–∞_ ratio only slightly above the bioequivalence range, which defines bioequivalence as an exposure ratio for AUC and C_max_ within a range of 0.8–1.25 (European Medicines Agency, 2010). These findings therefore suggest that co-administered OATP1B substrates such as most statins, but also, for example, repaglinide or bosentan, can be safely combined with bulevirtide (Niemi, 2007). It can also be assumed, that the substantial changes by bulevirtide in plasma concentration of endogenous OATP1B substrates such as bile acids do not trigger drug interactions with OATP1B. However, in patients using high statin doses and where concomitant factors, potentially increasing statin exposure, are present, caution may be required when using bulevirtide.

We were able to include a small number of carriers of polymorphic SLCO1B1 alleles/haplotypes. Two of these had a genotype with expected lower transport function. Similar to various pharmacogenetic studies (Niemi et al., 2004; Ho et al., 2007; Nakanishi and Tamai, 2012), pravastatin AUC and C_max_ were higher in those two participants (with the haplotypes SCLO1B1*5/*15 and *15/*15) than in homozygous or heterozygous carriers of wild-type alleles.

While this genetically reduced OATP1B1 activity is associated with statin-induced myotoxicity in patients with *5 or *15 haplotypes (Group et al., 2008), our SCLO1B1*5/*15 and *15/*15 carriers had 11%–28% lower pravastatin AUC values during bulevirtide, which was in contrast to our findings in the wild-type group (with the strongest increase in pravastatin exposure in the homozygous wild-type group). Unfortunately, we were not able to find further carriers of these genotypes, therefore, it remains unclear how to interpret the findings in these individuals. Nevertheless, taken together this supports the theory that in volunteers with already reduced OATP1B1 function, drug-related OATP1B1 inhibition may not be relevant.

As expected, bid administration of bulevirtide led to a lower C_max_ compared to the 10 mg once-daily administration (285 ng/ml *versus* 423 ng/ml) (Blank et al., 2018). Interestingly, the twice daily 5 mg dosing of bulevirtide showed a significant 1.32 higher total AUC over 24 h as compared to the 10 mg daily regimen (Blank et al., 2018). In the pharmacokinetic model for bulevirtide, the modelled s. c. 10 mg dose provided a target saturation of over 80% for at least 15 h (Blank et al., 2016). The overall higher exposure of bulevirtide with concurrently lower C_max_ values is expected to provide a longer saturation of NTCP for the blockade of virus entry with a potentially even better tolerability profile for the drug. Given the situation that *in vitro* bulevirtide inhibits HDV infection in sub-nanomolar ranges with an EC_50_ of 669 pM, whereas the bile acid transport is in comparable conditions only inhibited at 52.2 nM, it might well be possible to even decrease the bulevirtide dose further with preserved antiviral function (Nkongolo et al., 2014). The bid dosing would then still provide a longer duration for target occupation which might be favorable. However, any bid application of bulevirtide compared to a daily administration is inconvenient for patients and may lower adherence.

In a previous trial administering once-daily 10 mg bulevirtide for 10 d (Blank et al., 2018) the eCl_met_ of midazolam decreased to 0.71. However, in this previous trial, bulevirtide was co-administered with tenofovir and it was impossible to differentiate between the influence of bulevirtide and the influence of tenofovir. Therefore, in the present trial we aimed to evaluate the bulevirtide effect on eCl_met_ of midazolam as a marker for CYP3A4 activity and found that CYP3A4 activity remained essentially unchanged with a very small, clinically irrelevant 1.17 increase of eCl_met_ at one time point at bulevirtide steady state, suggesting that CYP3A4 substrates are not affected by bulevirtide to any meaningful extent. Concurrently, this trial confirmed that pravastatin does not modify CYP3A activity.

The tolerability of the still novel drug bulevirtide in this trial was completely in line with the excellent safety profile known so far. The injection site reactions attributed to bulevirtide have been described previously and were all transient (Bogomolov et al., 2016; Blank et al., 2018; European Medicines Agency, 2020).

The main limitation of the present trial is that healthy participants and not patients were studied. Therefore, the results may not be completely transferable to patients suffering from comorbidities, such as impaired liver and/or kidney function. Also due to the low sample number conclusions for carriers of known dysfunctional SLCO1B1 cannot be drawn.

In conclusion, this trial demonstrated, that the interaction of the novel HBV/HDV entry inhibitor bulevirtide with the drug transporter OATP1B is mild and no clinically relevant impairment of CYP3A4 activity has to be expected. The study suggests that OATP1B substrate drugs as well as CYP3A4 substrates may safely be used without dose adjustment in patients treated with bulevirtide. Only if patients require high statin doses and concomitant factors may potentially further increase statin exposure, close observation may be required when using bulevirtide. The general precautionary warning in the current EMA Summary of Product Characteristics against concomitant use of OATP1B1 substrates should be reconsidered and modified in order not to unnecessarily withdraw HDV patients from other important drugs, that are used, for example, for cardiovascular prevention.

## Data Availability

The raw data supporting the conclusion of this article will be made available by the authors, without undue reservation.
