# Supplementary material for: Evaluation of the drug-drug interaction potential of the novel hepatitis B and D virus entry inhibitor bulevirtide at OATP1B in healthy volunteers
Source: Front Pharmacol. 2023 Apr 6;14:1128547. doi: 10.3389/fphar.2023.1128547 (PMC10117888; doi:10.3389/fphar.2023.1128547)
Supplement: Supplementary file 1 [file DataSheet1.PDF]

Supplementary Material

Evaluation of the drug–drug interaction potential of the novel hepatitis B and D virus entry inhibitor bulevirtide at OATP1B in healthy volunteers

1 Inclusion and exclusion criteria

Inclusion criteria:

1. Age 18-50 years inclusive at the time of consent,
2. Males and females of child-bearing potential who are willing to use a highly effective method of contraception during the treatment and for 3 months after last administration of the investigational medicinal product (IMP) or women not of child-bearing potential (WNCBP) or individuals who are convincingly sexually abstinent,
3. Participation in a genotyping study (K093) to determine OATP genotypes,
4. Understanding, ability, and willingness to fully comply with trial interventions and restrictions, and
5. Ability to provide written, personally signed and dated informed consent to participate in the trial, in accordance with the International Conference on Harmonisation (ICH) Good Clinical Practice (GCP) Guideline E6, and applicable regulations, prior to any trial-related interventions.

Exclusion criteria at the time of screening:

1. Clinically significant or relevant abnormalities in the medical history, physical examination, and laboratory evaluation as assessed by the investigator,
2. Any medical disorder that may require treatment or make the participant unlikely to fully complete the trial, or any condition that presents undue risk from the IMP or trial interventions,
3. Pregnancy or breast feeding,
4. Clinically relevant ongoing or clinically relevant history of physical or psychiatric illness as judged by the investigator,
5. Any acute or chronic illness or clinically relevant finding known or expected to modify absorption, distribution, metabolism, or excretion of pravastatin and bulevirtide,

6. History of cholecystectomy,
7. Any known history of severe allergic or anaphylactic reactions to drugs or food or any other clinically significant allergies (except mild forms of hay fever),
8. Any known allergies to the compound or additives of pravastatin, midazolam, or bulevirtide,
9. History of any form of hepatitis (except for hepatitis A) as detected by hepatitis screening tests, any chronic liver disease or nutritive-toxic liver injury,
10. A positive human immunodeficiency virus (HIV) antibody screen,
11. A positive result in the drug screening test at SCR, Clinically relevant findings in any of the following investigations at SCR. Minor deviations of laboratory values from the normal range can be acceptable, if judged by the investigator to be of no clinical relevance for this trial.
    - Hemoglobin (Hb) < 12 g/dl (males) or < 11 g/dl (females),
    - Creatinine (Crea) clearance (Cl) < 60 mL/min/m^2 (Cockroft-Gault),
    - Bilirubin > upper limit of normal (ULN) x 1.2, In case of suspected Gilbert's disease: non-fasting total bilirubin ≤ ULN x 1.2 and fasting total bilirubin ≤ ULN x 1.5 are acceptable.
    - Alanine aminotransferase (ALT) > ULN x 1.1,
    - Aspartate aminotransferase (AST) > ULN x 1.2,
    - Creatine kinase (CK) not within normal limits (Volunteers with CK elevations between ULN and ULN x 3 may be included if troponin T is negative), and
    - Thyroid stimulating hormone (TSH) not within normal limits,
13. A history of a prior CK elevation under a statin therapy,
14. Any inherited muscular disease or any family history of inheritable muscular disease,
15. Use of any medication (prescription medication, non-prescription medication, or over-the-counter medicine including multivitamin, herbal, or homeopathic preparations) within 7 days (d) prior to the expected date of first dose of IMP except of hormonal contraception,
16. Consumption of citrus fruits or products of these fruits within 7 d prior to the expected date of first dose of IMP and expected noncompliance to refrain from the products until visit 9 of this trial,
17. Male participants who consume more than 21 units of alcohol per week or 3 units per d. Female participants who consume more than 14 units of alcohol per week or 2 units per d and expected noncompliance to refrain from alcohol 24 hours (h) prior to visit 1 until visit 9 of this trial,

18. Any intake of substances known to induce or inhibit drug metabolizing enzymes or transport enzymes within a period of less than 5 times the respective elimination half-life ($t_{1/2}$) with regard to the expected date of first dose of IMP, and
19. Use of an IMP within 30 d prior to the expected date of receiving the first dose of IMP or active enrolment in another drug or vaccine clinical trial.

Exclusion criteria at trial day 1, prior to dosing:

20. Use of any medication (prescription medication, non-prescription medication, or over-the-counter medicine including multivitamin, herbal, or homeopathic preparations) within 7 d prior to the first dose of IMP except of hormonal contraception,
21. Consumption of citrus fruits or products of these fruits within 7 d prior to the expected date of first dose of IMP and expected noncompliance to refrain from the products until visit 9 of this trial,
22. Alcohol intake within 24 h prior to receiving the trial drug,
23. Any intake of substances known to induce or inhibit drug metabolizing enzymes or transport enzymes within a period of less than 5 times the respective elimination $t_{1/2}$ with regard to the expected date of first dose of IMP, and
24. Use of an IMP within 30 d prior to receiving the first dose of IMP or active enrolment in another drug or vaccine clinical trial.

2 Results

Supplementary Table 1: Pharmacokinetics of a 40-mg single oral dose of pravastatin alone and at bulevirtide steady state in all participants, in genotype groups, and in individuals by SLCO1B1 genotype.

**Pharmacokinetic parameter**	**Pravastatin**	
	**baseline**	**at bulevirtide steady state**
**All participants (N = 19)**		
$AUC_{0-\infty}$ (h*ng/mL) [a]	207 (161; 267)	273 (214; 348)
Geometric mean ratio[a]		*1.32 (1.08; 1.62)*
		p = 0.03
C_{max} (ng/ml] [a]	91.4 (67.8; 123)	121 (89.8; 163)
Geometric mean ratio[a]		*1.32 (1.01-1.73)*
		p = 0.02
**All participants without *5/*15 and *15/*15**		
$AUC_{0-\infty}$ (h*ng/mL) [a]	191 (148; 245)	267 (203; 350)
Geometric mean ratio [a]		*1.4 (1.13; 1.73)*
		p = 0.02
C_{max} (ng/ml] [a]	83.1 (58.4; 118)	122 (85.3;176)
Geometric mean ratio [a]		*1.47 (1.03; 2.11)*
		p = 0.07
**Genotypes *1A/*1A, homozygous wildtype (N = 8)**		
$AUC_{0-\infty}$ (h*ng/mL) [a]	196 (119; 324)	344 (261; 453)
Geometric mean ratio [a]		*1.76* (1.17; 2.64)
		p = 0.03
C_{max} (ng/ml] [a]	84.9 (46.8; 154)	160 (110;232)
Geometric mean ratio [a]		*1.88 (1.11; 3.20)*
		p = ns
**Genotypes *1A/*1B, heterozygous wildtype (N = 5)**		
$AUC_{0-\infty}$ (h*ng/mL) [a]	169 (116; 247)	186 (96.1; 362)
Geometric mean ratio [a]		*1.10* (0.75; 1.61)
		p = ns
C_{max} (ng/ml] [a]	80.4 (51.2; 126)	80.1 (38.0;169)
Geometric mean ratio [a]		*1.00 (0.68; 1.46)*
		p = ns
**Genotypes *1A/*1A, *1A/*1B, homozygous and heterozygous wildtype (N = 13)**		
$AUC_{0-\infty}$ (h*ng/mL) [a]	185 (137; 250)	272 (200; 369)
Geometric mean ratio [a]		*1.47* (1.11; 1.94)
		p = 0.03
C_{max} (ng/ml] [a]	83.1 (58.4; 118.21)	122 (85.3; 176)
Geometric mean ratio [a]		*1.88*
		p = ns

**Genotypes homozygous or heterozygous *1B/ *5/ *15**		
**Genotype *5/*15**		
$AUC_{0-\infty}$ (h*ng/mL) [a]	331	294
Ratio		*0.89*
C_{max} (ng/ml] [a]	143	98.3
Ratio		*0.69*
**Genotype *15/*15**		
$AUC_{0-\infty}$ (h*ng/mL) [a]	532	382
Ratio		*0.72*
C_{max} (ng/ml] [a]	316	166
Ratio		*0.52*
**Genotype *1B/*15**		
$AUC_{0-\infty}$ (h*ng/mL) [a]	197	207
Ratio		*1.05*
C_{max} (ng/ml] [a]	55.0	78.9
Ratio		*1.43*
**Genotype *1B/*5[b]**		
$AUC_{0-\infty}$ (h*ng/mL) [a]	197	265
Ratio		*1.35*
C_{max} (ng/ml] [a]	72.6	120
Ratio		*1.65*
**Genotype *1B/*1B**		
$AUC_{0-\infty}$ (h*ng/mL) [a]	412	609
Ratio		*1.48*
C_{max} (ng/ml] [a]	186	370
Ratio		*1.99*
**Genotype *1B/*1B**		
$AUC_{0-\infty}$ (h*ng/mL) [a]	119	118
Ratio		*0.99*
C_{max} (ng/ml] [a]	59.7	45.9
Ratio		*0.77*

$AUC_{0-\infty}$, area under the concentration-time curve extrapolated from time 0 to infinity; C_{max}, maximum concentration;

[a] Pharmacokinetic parameter are shown as geometric means with 95 % confidence interval. Geometric mean ratios for AUC and C_{max} are shown with 90 % confidence interval ($p < 0.1$ considered statistically significant). (European Medicines Agency, 2010).

[b] Based on genotype determination method, genotype cannot be differentiated from *1A/*15 genotype.

Supplementary Table 2. $AUC_{0–12}$ of unconjugated, glycine-conjugated, taurine-conjugated, and total bile acids at baseline, at bulevirtide steady state, and at bulevirtide steady state with pravastatin (n = 10).

**$AUC_{0–12}$[a] [µmol/l*min]**	**Baseline**	**With bulevirtide at steady state**	**With bulevirtide at steady state + pravastatin**
Unconjugated	405 (251; 654)	1468* (933; 2308)	1240* (778; 1978)
Ratio[b]		3.62	3.06
Glycine-conjugated	2181 (1492; 3189)	82863** (58362; 117650)	88097** (65891; 117787)
Ratio[b]		38.0	40.4
Taurine-conjugated	313 (205; 480)	19263** (12717; 29179)	19847** (14805; 26605)
Ratio[b]		61.5	63.3
Total bile acids	2957 (2022; 4324)	104176** (72953; 148763)	109660** (82405; 145929)
Ratio[b]		35.2	37.1

[a] Geometric means with 95% CI

[b] vs. baseline

*p = 0.001 vs. baseline.

**p < 0.0001 vs. baseline.

Supplementary Table 3. Adverse events (according to MedDRA, lower level term) by causality, sorted by frequency.

Adverse Events	Frequency
Causality: definitely related	
Administration site reaction	15
Causality: possibly related	
Headache	4
Hypotension	3
Alanine aminotransferase increase grade 1	2
Aspartate aminotransferase increase grade 1	2
Lymphocyte count decrease grade 1	2
Amylase increase grade 1	1
Constipation	1
Creatine phosphokinase increase grade 1	1
Dyspepsia	1
Ear pain	1
Erythema	1
Feeling cold	1
Hypertension	1
Lipase increase grade 1	1
Lipase increase grade 2	1
Malaise	1
Nausea	1
Oropharyngeal pain	1
Rhinitis	1
Upper abdominal pain	1
Upper respiratory tract infection	1
Causality: not related	
Headache	4
Arthropod bite	1
Dizziness	1
Hemoglobin decrease	1
Menstrual discomfort	1
Metrorrhagia	1
Oropharyngeal pain	1
Premenstrual cramps	1
Skin irritation	1
Upper abdominal pain	1
Upper respiratory tract infection	1
Urinary tract infection	1
Xeroderma	1

3 References

European Medicines Agency (2010). Guideline on the investigation of bioequivalence. *https://www.ema.europa.eu/en/documents/scientific-guideline/guideline-investigation-bioequivalence-rev1_en.pdf.* [Accessed 16 December 2022].
